# Clusters of anthropometric indicators of body fat associated with maximum oxygen uptake in adolescents

**DOI:** 10.1371/journal.pone.0193965

**Published:** 2018-03-13

**Authors:** Eliane Cristina de Andrade Gonçalves, Heloyse Elaine Gimenes Nunes, Diego Augusto Santos Silva

**Affiliations:** 1 Research Center in Kinanthropometry and Human Performance, Federal University of Santa Catarina, Florianópolis, Santa Catarina, Brasil; 2 Study Lab of Striated Muscle, Federal University of Mato Grosso do Sul, Campo Grande, Mato Grosso do Sul, Brasil; The Chinese University of Hong Kong, HONG KONG

## Abstract

**Introduction:**

The aim of this study was to evaluate different clusters of anthropometric indicators (body mass index | BMI |, waist circumference | WC |, waist-to-height ratio | WHtR |, triceps skinfold |TR SF|, subscapular skinfold |SE SF|, sum of the triceps and subscapular skinfolds | ΣTR + SE |, and sum of the triceps, subscapular and suprailiac folds | ΣTR + SE + SI|) associated with the VO_2_max levels in adolescents.

**Methods:**

The study included 1,132 adolescents (aged 14–19 years) enrolled in public schools of São José, Santa Catarina, Brazil, in the 2014 academic year. The dependent variable was the cluster of anthropometric indicators (BMI, WC, WHtR, TR SF, SE SF, SI SF, ΣTR + SE and ΣTR + SE + SI) of excess body fat. The independent variable was maximum oxygen uptake (VO_2_max), estimated by the modified Canadian aerobic fitness test—mCAFT. Control variables were: age, skin color, economic level, maternal education, physical activity and sexual maturation. Multinomial logistic regression was used for associations between the dependent and independent variables. Binary logistic regression was performed to identify the association between adolescents with all anthropometric indicators in excess and independent variables.

**Results:**

One in ten adolescents presented all anthropometric indicators of excess body fat. Multinomial regression showed that with each increase of one VO_2_max unit, the odds of adolescents having three, four, five or more anthropometric indicators of excess body fat decreased by 0.92, 0.85 and 0.73 times, respectively. In the binary regression, this fact was reconfirmed, demonstrating that with each increase of one VO_2_max unit, the odds of adolescents having simultaneously the eight anthropometric indicators of excess body fat decreased by 0.55.

**Conclusion:**

It was concluded that with each increase of one VO_2_max unit, adolescents decreased the odds of simultaneously presenting three or more anthropometric indicators of excess body fat, regardless of biological, economic and lifestyle factors. In addition, the present study identified that one in ten adolescents had all anthropometric indicators of excess body fat.

## Introdution

Anthropometry is one of the most widely used ways to evaluate body composition in epidemiological studies, since it is considered to be easy to apply, low cost and with good indexes of validity when compared to more precise methods [1]. In this sense, each anthropometric indicator provides specific information on body composition [1]. Body mass index (BMI) is considered an indicator of total body fat, skin folds indicate body fat distribution, waist circumference (WC) and waist-to-height ratio (WHtR) are indicators of central adiposity [1]. However, when using only one of these anthropometric indicators, it is possible that the limitations of these measures would hinder the generalization of results, suggesting the need to use more than one anthropometric indicator to assess body fat as a complementary form of information [2].

In addition to body composition being a strong indicator of health in adolescence, aerobic fitness is another component with good predictive health capacity in all life cycles [3]. The literature is concise in reporting that higher amounts of body fat are related to lower maximal oxygen uptake (VO_2_max) and the greater amount of muscle mass is associated with higher VO_2_max values [3,4]. However, evidence of how different patterns of body fat distribution may interfere with VO_2_max levels is unclear. From the analysis of the relationship between body fat distribution and VO_2_max, it is possible to infer how fat localization interferes with performance in aerobic fitness tests, helping in the elaboration of strategies for maintenance and increase of VO_2_max levels [4].

Previous studies have analyzed clusters of some anthropometric indicators (BMI, WC, sum of five skinfolds and WHtR) [2]. However, no study was found associating eight anthropometric indicators (BMI, WC, WHtR, triceps skinfold | TR SF |, subscapular fold | SE SF | suprailiac fold | SI SF | sum of triceps and subscapular folds | ΣTR + SE |, and sum of triceps, subscapular and suprailiac skinfolds | ΣTR + SE + SI|) simultaneously, thus presenting the influence of the different localities of body fat on VO_2_max, which justifies this study.

The grouping of anthropometric indicators demonstrates the simultaneity of health risk conditions related to the excessive amount of fat in different parts of the body, and this fact, added to the lower VO_2_max values, increases the chances of adolescents presenting insulin resistance, metabolic syndrome and consequently higher morbidity rates and early mortality [5]. Moreover, excess body fat and VO_2_max are considered modifiable factors, which allow the development of actions related to increases in VO_2_max values, considering that VO_2_max is able to significantly reduce fat tissue [5].

Therefore, the aim of the present study was to evaluate different clusters of anthropometric indicators (BMI, WC, WHtR, TR SF, SE SF, SI SF, ΣTR + SE and ΣTR + SE + SI) associated with VO_2_max levels in adolescents.

## Materials and methods

This cross-sectional study was approved by the Human Research Ethics Committee of the Federal University of Santa Catarina (UFSC) under CAAE protocol: 33210414.3.0000.0121.

The study population consisted of 5,182 high school students aged 14–19 years enrolled in public high schools of the city of São José, southern Brazil, distributed in 11 eligible schools and 170 high school classes.

The sampling process was determined in two stages: 1) stratified by public high schools (n = 11); 2) clusters of classes considering shift and school year (n = 170). In the second stage, all high school students who were in the classroom on the days of data collection were invited to participate in the research. The probabilistic sample consisted of 1,132 adolescents. Data on estimates for the calculation of the sampling process (inclusion, exclusion criteria and eligibility) can be found in literature [6]. Collection was carried out in the second half of 2014 during the months of August to November. The research team was composed of undergraduate and graduate students previously familiar and trained to apply questionnaires and physical assessments.

### Dependent variable

The dependent variable was the simultaneity of anthropometric indicators of excess body fat. Eight anthropometric indicators of body fat were analyzed: BMI, WC, WHtR, TR SF, SE SF, SI SF, ΣTR + SE and ΣTR + SE + SI.

Anthropometric data on body mass, height, WC, and skinfolds were measured according to International Society for the Advancement of Kinanthropometry (ISAK) procedures by a single level 1 ISAK-certified assessor. For the measurements of skinfolds, Cescorf adipometer (Porto Alegre, Brazil) with precision of 0.1mm was used.

WC was measured with an inelastic Sanny metallic anthropometric tape with 0.1 mm resolution (São Paulo, Brazil), measured at the lowest perimeter between the last rib and the superior border of the iliac crest. WHtR was evaluated by the relationship between WC (cm) and height (cm). BMI was calculated by the relationship between body mass (kilograms) and height in squared meters.

In the present study, all anthropometric indicators were classified as having as cutoff value the 75^th^ percentile of the sample distribution, considering that values above the 75^th^ percentile are sensitive in the body fat diagnosis in children and adolescents [7]. Thus, values equal to or greater than the 75^th^ percentile (P≥75) of anthropometric indicators of body fat were considered high because they represented the quartile with the highest percentage and for the cutoff points of all the anthropometric indicators to be equated. Score ranging from 0 (no anthropometric indicators of excess) to 8 (simultaneous presence of eight anthropometric indicators of excess) was used to classify individuals as for the simultaneity of anthropometric indicators.

### Independent variable

Aerobic fitness was estimated using the modified Canadian Aerobic Fitness test—mCAFT [8], validated in comparison with indirect calorimetry in men and women aged 15–69 years [9]. To perform the test, adolescents had to complete one or more stages of three minutes each, in which they had to climb up and down two steps of 20.3 centimeters each. The stage and the initial velocity were predetermined according to sex and age. The rhythm to perform the steps within each stage of the test was done by musical cadence, indicating the moment when the adolescent should go up and down the step [8]. The test was finalized only when the subject reached 85% of maximal heart rate (determined by the 220-age formula) [8], which was measured using the Polar brand H7 Bluetooth Frequencmeter (Kempele, Finland). If the subject did not reach 85% of the maximum heart rate in a given stage, a new stage was started shortly after the end of the last one, until 85% of the maximum heart rate was reached for the end of the test. The final stage of the test was the stage in which the adolescent was able to perform completely. That is, if 85% of the maximum heart rate was reached during a given stage, the previous stage was recorded as the final stage.

Oxygen expenditure during exercise performed by the adolescent and reference values for the determination of the beneficial health zone for aerobic fitness are determined by the Canadian battery [8]. The equation of the aerobic fitness score determined by the Canadian battery is: Score = 10 [17.2 + (1.29 x Oxygen Expenditure)—(0.09 x weight in kg)—(0.18 x age in years). The result was divided by 10 to obtain the value estimated for the VO_2_max of adolescents [8], which was continuously analyzed.

### Control variables

Sociodemographic variables were collected through a self-administered questionnaire. Skin color was self-reported according to the Brazilian Institute of Geography and Statistics [10] and dichotomized in "White" and "Black / brown / yellow / indigenous". Age was categorized as "14–16 years" and "17–19 years". Economic level was estimated by the questionnaire of the Brazilian Association of Research Companies [11] and dichotomized into "High" ("A1", "A2", "B2") and "Low" (“C1”; “C2”; “D”; “E”). Maternal schooling was divided into "≥ 8 years of study" and "<8 years of study".

Physical activity was analyzed by frequency of practice in the last seven days, for at least 60 minutes. Adolescents who practiced less than five times a week were considered little physically active. This parameter was used from evidence that demonstrated that it is necessary to practice 60 minutes of physical activity in at least five times a week for health maintenance in adolescence [12].

Sexual maturation was evaluated according to criteria proposed by Tanner [13], validated and reproducible in the Brazilian population [14]. Stages were indicated by self-assessment (figures) of breast (female) and genital (male) development after individual and previous explanation of the instrument by the researcher, always of the same sex as the adolescent. Due to the low frequency of adolescents in the pre-pubertal stage (0.2%), categories were "Pre-pubertal / pubertal" and "Post-pubertal".

## Statistical analysis

In the descriptive analysis of variables, means, standard deviations and frequency distribution were used. Data normality of continuous variables was verified by analyzing asymmetry and kurtosis. Considering that all kurtosis and asymmetry values were close to zero, data were considered normal [15,16]. The effect size was calculated according to literature [17].

The chi-square test of heterogeneity and linear tendency was used to evaluate differences between groups for each anthropometric indicator of body fat. The combinations among anthropometric indicators were presented and the ratio between observed and expected prevalence (O / E) was calculated [18]. The observed prevalence was identified for the sample of the present study, and the expected prevalence was calculated by multiplying the individual probability of each anthropometric indicator of body fat based on its occurrence in the study population. Thus, it was possible to identify combinations that are above or below expected [18].

The associations between the dependent variable "simultaneity of anthropometric indicators of excess body fat" and independent variables were analyzed by multinomial logistic regression, with estimates of odds ratio (OR) and confidence intervals (95% CI). The reference category was "zero anthropometric indicator of excess body fat". Adolescents who presented six (3.8%), seven (5.1%) and eight (12.0%) anthropometric indicators of excess body fat were classified in the category "five or more anthropometric indicators of excess body fat" due to the low frequency.

Binary logistic regression was performed with estimation OR and respective 95% CI to identify the association between adolescents who presented "eight anthropometric indicators of excess body fat" and independent variables. The reference category was "zero anthropometric indicator of excess body fat". The adjusted analysis was controlled by all independent variables. For all statistical tests, p <0.05 was used and the SPSS 21.0 software was used.

## Results

A total of 1,132 adolescents with mean VO_2_max of 38.80 ± 5.83mL / kg / min were analyzed (Table 1). The majority of individuals were female aged 14–16 years, white skin color, whose mothers had less than eight years of schooling, high economic level, little physically active and pre-pubertal / pubertal (Table 2).

**Table 1:** Total and stratified by sex of mean and standard deviation of age, height, body mass, anthropometric indicators and VO_2_max of adolescents.

Variables	Total sample	Males	Females	p-value	Cohen’s d
	M±SD	M±SD	M±SD		
**Age (years)**	16.22±1.14	16.28±1.19	16.16±1.10	0.15	0.10
**Height (cm)**	166.56±8.81	172.59±7.35	161.17±6.09	<0.01	1.69
**Body mass (kg)**	61.67±12.20	65.43±12.07	58.31±11.32	0.25	0.60
**BMI (kg/m**^**2**^**)**	22.16±3.72	21.89±3.44	22.41±3.95	0.25	0.14
**WC (cm)**	71.48±8.03	73.79±7.71	69.41±7.74	<0.01	0.56
**WHtR (cm)**	0.42±0.04	0.42±0.04	0.43±0.04	0.51	0.25
**TR SF (mm)**	14.94±7.34	10.75±5.13	18.70±6.99	<0.01	1.29
**SE SF (mm)**	13.32±6.73	10.76±4.86	15.60±7.33	<0.01	0.77
**SI SF (mm)**	16.25±10.36	12.85±8.45	19.22±10.94	<0.01	0.65
**ΣTR+SE (mm)**	28.26±13.49	21.51±9.53	34.30±13.66	<0.01	1.08
**ΣTR+SE+SI (mm)**	41.34±25.29	32.25±19.06	49.25±27.33	<0.01	0.72
**VO**_**2**_**max (ml/kg/min)**	38.80±5.83	42.68±5.34	35.33±3.66	<0.01	1.60

M: mean; SD: standard deviation; BMI: body mass index; WC: Waist circumference; WHtR: Waist to height ratio; TR SF: triceps skinfold; SE SF: subscapularis skinfold; SI SF: suprailiac skinfold; ΣTR + SE: sum of triceps and subscapularis skinfolds; ΣTR + SE + SI: sum of triceps, subscapular and suprailiac skinfolds; VO_2_max: maximum oxygen uptake; p≤0.05 (Student’s t test).

**Table 2:** Characteristics of anthropometric indicators of adolescents according to demographic and economic variables.

Variables	Total sample		BMI high		WC high		WHtR high		TR SF high			SE SF high		SI SF high		ΣTR+SE high		ΣTR+SE+SI high	
	n	(%)	n	(%)	n	(%)	n	(%)	n		(%)	n	(%)	n	(%)	n	(%)	n	(%)
**Total**	1132		1008	(25.1)	930	(25.1)	864	(32.3)	931		(28.2)	931	(28.1)	930	28.2	931	28,2	929	28,1
**Sex**				p = 0.96		p = 0.97		p = 0.16			p = 0.85		p = 0.79	p = 0.80		p = 0.85		p = 0,76	
Males	519	(45.8)	119	(47.0)	110	(47.2)	141	(50.5)	123		(46.8)	122	(46.6)	122	(46.6)	123	(46,8)	121	(46,4)
Females	613	(54.2)	134	(53.0)	123	(52.8)	138	(49.5)	140		(53.2)	140	(53.4)	140	(53.4)	140	(53,2)	140	(53,6)
**Age**				p = 0.75		**p = 0.04**		**p = 0.01**			p = 0.42		p = 0.10	p = 0.98		p = 0.70		p = 0,10	
14–16 years	677	(59.8)	145	(57.3)	122	(52.4)	144	(51.6)	158		(60.1)	141	(53.8)	152	(58.0)	150	(57,0)	150	(57,5)
17–19 years	455	(40.2)	108	(42.7)	111	(47.6)	135	(48.4)	105		(39.9)	121	(46.2)	110	(42.0)	113	(43,0)	111	(42,5)
**Skin color**				p = 0.52		p = 0.07		p = 0.20			p = 0.73		p = 0.24	p = 0.75		p = 0.29		p = 0,41	
White	691	(61.8)	151	(61.4)	131	(57.7)	162	(59.8)	158		(61.7)	152	(59.6)	159	(61.9)	154	(59,9)	154	(60,4)
Brown/Black/Yellow/Indigenous	427	(38.2)	95	(38.6)	96	(42.3)	109	(40.2)	98		(38.3)	103	(40.4)	98	(38.1)	103	(40,1)	101	(39,6)
**Maternal education**				p = 0.76		p = 0.59		p = 0.60			p = 0.88		p = 0.50	p = 0.26		p = 0.71		p = 0,65	
≥ 8 years of schooling	487	(43.0)	112	(25.8)	105	(26.3)	125	(33.6)	112		(27.9)	118	(29.4)	115	(28.7)	116	(28,9)	116	(28,9)
≤ 8 years of schooling	631	(56.4)	140	(25.0)	127	(24.5)	153	(31.7)	148		(28.6)	141	(27.2)	146	(28.2)	144	(27,8)	142	(27,5)
**Socioeconomic level**				p = 0.42		p = 0.70		p = 0.43			p = 0.54		p = 0.37		p = 0.55		p = 0,37		p = 0,13
High	666	(69.5)	142	(66.4)	135	(67.2)	157	(66.0)	152		(66.7)	149	(65.9)	154	(66.7)	149	(65,9)	148	(64,3)
Low	292	(30.5)	72	(33.6)	66	(32.8)	81	(34.0)	76		(33.3)	77	(34.1)	77	(33.3)	77	(34,1)	82	(35,7)
**Physical activity**				p = 0.36		p = 0.44		p = 0.92			p = 0.20		p = 0.10	p = 0.07		p = 0.21		p = 0,23	
Physically active	252	(22.8)	63	(25.5)	58	(25.4)	65	(24.2)	53		(20.8)	50	(19.9)	50	(19.6)	53	(20,9)	53	(21,0)
Little physically active	851	(77.2)	184	(74.5)	170	(74.6)	204	(75.8)	202		(79.2)	201	(80.1)	205	(80.4)	201	(79,1)	199	(79,0)
**Sexual maturation**				**p<0.00**		**p<0.00**		**p = 0.00**			**p = 0.02**		**p = 0.01**		**p = 0.00**		**p = 0,00**		**p = 0,00**
Pre-pubertal / pubertal	802	(71.4)	153	(61.0)	143	(62.2)	176	(63.8)	171		(65.8)	168	(65.1)	168	(64.9)	166	(63,8)	162	(62,8)
Post-pubertal	321	(28.6)	98	(39.0)	87	(37.8)	100	(36.8)	89		(34.2)	90	(30.0)	91	(35.1)	94	(36,2)	96	(37,2)

BMI: body mass index; WC: Waist circumference; WHtR: Waist to height ratio; TR SF: triceps skinfold; SE SF: subscapularis skinfold; SI SF: suprailiac skinfold; ΣTR + SE: sum of triceps and subscapularis skinfolds; ΣTR + SE + SI: sum.

Individuals aged 14–16 years had higher prevalence of high WC and WHtR. Pre-pubertal / pubertal adolescents presented higher prevalence of excess adiposity for all anthropometric indicators of body fat (Table 2).

There was a significant difference in the means of all anthropometric indicators of body fat when compared the values of the anthropometric indicators considered normal and those in excess (Table 3).

**Table 3:** Values of mean and standard deviation of VO_2_max according to the anthropometric indicators of adolescents.

	VO_2_max (ml/kg/min)		
	M±SD	p-value	Cohen’s d
**BMI**		<0.01	0.50
Normal	39.52±5.78		
High	36.67±5.45		
**WC**		<0.01	0.60
Normal	39.59±5.83		
High	36.41±5.13		
**WHtR**		<0.01	0.57
Normal	39.50±5.66		
High	37.36±5.97		
**TR SF**		<0.01	
Normal	39.81±5.89		0.60
High	36.22±5.89		
**SE SF**		<0.01	
Normal	39.87±5.87		0.66
High	36.07±4.75		
**SI SF**		<0.01	
Normal	39.91±5.89		0.71
High	36.01±4.64		
**ΣTR + SE**		<0.01	
Normal	39.95±5.91		0.77
High	35.90±4.48		
**ΣTR + SE + SI**		<0.01	0.73
Normal	39.91±5.85		
High	36.00±4.77		

M: mean; SD: standard deviation; BMI: body mass index; WC: Waist circumference; WHtR: Waist to height ratio; TR SF: triceps skinfold; SE SF: subscapularis skinfold; SI SF: suprailiac skinfold; ΣTR + SE: sum of triceps and subscapularis skinfolds; ΣTR + SE + SI: sum of triceps, subscapular and suprailiac skinfolds; VO_2_max: maximum oxygen uptake; p≤0.05 (Student’s t test).

From the total individuals investigated, 12% presented excess body fat for all eight anthropometric indicators, and this was 4,000 times higher than expected in the sample. When analyzing the simultaneous presence of excess adiposity of seven anthropometric indicators (BMI, WC, WHtR, SE SF, SI SF, ΣTR + SE and ΣTR + SE + SI), prevalence of approximately 267 times greater than expected was observed. When WHtR, TR SF, SE SF, SI SF, ΣTR + SE and ΣTR + SE + SI indicators were combined, the observed prevalence was 31 times higher than expected (0.032%). In relation to five anthropometric indicators (TR SF, SE SF, SI SF, ΣTR + SE and ΣTR + SE + SI), the prevalence observed was 22 times higher than expected. For the four anthropometric indicators, the prevalence of the combination of TR SF, SE SF, ΣTR + SE and ΣTR + SE + SI presented a higher O / E ratio (5.263%). For three anthropometric indicators (BMI, WC and WHtR), the observed prevalence was 2.827 times greater than expected. The simultaneity between BMI and WHtR had higher observed prevalence (0.861 times greater than expected). The highest observed prevalence for one anthropometric indicator in excess (WHtR) was 0.9% higher than expected (Table 4).

**Table 4:** Prevalence of combinations of anthropometric indicators in adolescents.

										Prevalence		
Anthropometric indicators	BMI	WC	WHtR	TR SF	SE SF	SI SF	ΣTR + SE	ΣTR + SE + SI	n	Observed% (95%CI)	Espected% (95%CI)	O/E
**8**	**+**	**+**	**+**	**+**	**+**	**+**	**+**	**+**	111	12.000 (9.080–14.910)	0.003 (0.000–0.050)	4.000.000
**7**	**+**	**+**	**+**	**+**	**+**	**+**	**-**	**+**	01	0.100 (0.000–0.380)	0.009 (0.000–0.090)	11.111
	**+**	**+**	**+**	**+**	**+**	**-**	**+**	**+**	08	0.900 (0.050–1.740)	0.009 (0.000–0.090)	100
	**+**	**+**	**+**	**+**	**-**	**+**	**+**	**+**	06	0.700 (0.000–1.440)	0.009 (0.000–0.090)	77.777
	**+**	**+**	**+**	**-**	**+**	**+**	**+**	**+**	22	2.400 (1.020–3.770)	0.009 (0.000–0.090)	266.666
	**+**	**-**	**+**	**+**	**+**	**+**	**+**	**+**	04	0.400 (0.000–0.960)	0.010 (0.000–0.100)	40.000
	**-**	**+**	**+**	**+**	**+**	**+**	**+**	**+**	06	0.700 (0.000–1.440)	0.010 (0.000–0.100)	70.000
**6**	**+**	**+**	**+**	**+**	**+**	**-**	**+**	**-**	02	0.200 (0.000–0.600)	0.083 (0.000–0.340)	2.409
	**+**	**+**	**+**	**+**	**+**	**-**	**-**	**+**	01	0.100 (0.000–0.380)	0.023 (0.000–0.160)	4.347
	**+**	**+**	**+**	**+**	**-**	**-**	**+**	**+**	01	0.100 (0.000–0.380)	0.023 (0.000–0.160)	4.347
	**+**	**+**	**+**	**-**	**+**	**+**	**-**	**+**	02	0.200 (0.000–0.600)	0.023 (0.000–0.160)	8.695
	**+**	**+**	**+**	**-**	**+**	**-**	**+**	**+**	03	0.300 (0.000–0.790)	0.023 (0.000–0.160)	13.043
	**+**	**+**	**-**	**+**	**+**	**-**	**+**	**+**	02	0.200 (0.000–0.600)	0.019 (0.000–0.140)	10.526
	**+**	**+**	**-**	**+**	**-**	**+**	**+**	**+**	01	0.100 (0.000–0.380)	0.019 (0.000–0.140)	5.263
	**+**	**-**	**+**	**+**	**+**	**-**	**+**	**+**	04	0.400 (0.000–0.960)	0.027 (0.000–0.170)	14.814
	**+**	**-**	**-**	**+**	**+**	**+**	**+**	**+**	03	0.300 (0.000–0.790)	0.022 (0.000–0.150)	13.636
	**-**	**+**	**+**	**+**	**-**	**+**	**+**	**+**	01	0.100 (0.00–0.380)	0.027 (0.000–0.170)	3.703
	**-**	**+**	**+**	**-**	**+**	**+**	**+**	**+**	05	0.500 (0.000–1.130)	0.027 (0.000–0.150)	22.18
	**-**	**-**	**+**	**+**	**+**	**+**	**+**	**+**	09	1.000 (0.100–1.890)	0.032 (0.000–0.190)	31.250
**5**	**+**	**+**	**+**	**+**	**+**	**-**	**-**	**-**	01	0.100 (0.000–0.380)	0.059 (0.000–0.270)	1.694
	**+**	**+**	**+**	**+**	**-**	**-**	**+**	**-**	02	0.200 (0.000–0.600)	0.060 (0.000–0.170)	3.333
	**+**	**+**	**+**	**-**	**+**	**+**	**-**	**-**	01	0.100 (0.000–0.380)	0.059 (0.000–0.270)	1.694
	**+**	**+**	**+**	**-**	**-**	**+**	**-**	**+**	04	0.400 (0.000–0.960)	0.059 (0.000–0.270)	6.779
	**+**	**-**	**-**	**+**	**+**	**-**	**+**	**+**	01	0.100 (0.000–0.380)	0.057 (0.000–0.270)	1.754
	**+**	**-**	**+**	**-**	**+**	**+**	**-**	**+**	01	0.100 (0.000–0.380)	0.069 (0.000–0.300)	1.449
	**+**	**-**	**-**	**+**	**-**	**+**	**+**	**+**	02	0.200 (0.000–0.600)	0.057 (0.000–0.270)	3.508
	**-**	**+**	**+**	**+**	**+**	**-**	**+**	**-**	01	0.100 (0.000–0.380)	0.070 (0.000–0.300)	1.428
	**-**	**+**	**+**	**-**	**+**	**+**	**-**	**+**	03	0.300 (0.000–0.790)	0.069 (0.000–0.300)	4.347
	**-**	**-**	**+**	**+**	**+**	**-**	**+**	**+**	02	0.200 (0.000–0.600)	0.081 (0.000–0.330)	2.469
	**-**	**-**	**+**	**-**	**+**	**+**	**+**	**+**	01	0.100 (0.000–0.380)	0.081 (0.000–0.330)	1.234
	**-**	**-**	**+**	**+**	**-**	**+**	**+**	**+**	01	0.100 (0.000–0.380)	0.082 (0.000–0.330)	1.219
	**-**	**-**	**-**	**+**	**+**	**+**	**+**	**+**	14	1.500 (0.400–2.590)	0.067 (0.000–0.290)	22.388
**4**	**+**	**+**	**+**	**+**	**-**	**-**	**-**	**-**	02	0.200 (0.000–0.600)	0.152 (0.000–0.500)	1.315
	**+**	**+**	**+**	**-**	**+**	**-**	**-**	**-**	06	0.600 (0.000–1.290)	0.152 (0.000–0.500)	3.947
	**+**	**+**	**+**	**-**	**-**	**+**	**-**	**-**	01	0.100 (0.000–0.380)	0.152 (0.000–0.500)	0.657
	**+**	**-**	**+**	**+**	**-**	**-**	**+**	**-**	01	0.100 (0.000–0.380)	0.179 (0.000–0.550)	0.558
	**+**	**-**	**-**	**+**	**+**	**-**	**+**	**-**	02	0.200 (0.000–0.600)	0.144 (0.000–0.480)	1.388
	**+**	**-**	**+**	**-**	**+**	**+**	**-**	**-**	01	0.100 (0.000–0.380)	0.178 (0.000–0.550)	0.561
	**+**	**-**	**-**	**+**	**-**	**-**	**+**	**+**	01	0.100 (0.000–0.380)	0.146 (0.000–0.490)	0.684
	**+**	**-**	**-**	**-**	**+**	**+**	**-**	**+**	01	0.100 (0.000–0.380)	0.146 (0.000–0.480)	0.684
	**-**	**+**	**+**	**-**	**+**	**+**	**-**	**-**	01	0.100 (0.000–0.380)	0.178 (0.000–0.550)	0.561
	**-**	**+**	**-**	**+**	**+**	**-**	**+**	**-**	01	0.100 (0.000–0.380)	0.146 (0.000–0.490)	0.684
	**-**	**-**	**+**	**+**	**+**	**-**	**+**	**-**	01	0.100 (0.000–0.380)	0.209 (0.000–0.610)	0.478
	**-**	**-**	**+**	**-**	**+**	**+**	**-**	**+**	03	0.300 (0.000–0.790)	0.208 (0.000–0.610)	1.442
	**-**	**-**	**+**	**-**	**+**	**-**	**+**	**+**	01	0.100 (0.000–0.380)	0.208 (0.000–0.610)	0.480
	**-**	**-**	**-**	**+**	**+**	**-**	**+**	**+**	08	0.900 (0.050–1.740)	0.171 (0.000–0.540)	5.263
	**-**	**-**	**-**	**+**	**-**	**+**	**+**	**+**	07	0.800 (0.000–1.590)	0.172 (0.000–0.540)	4.651
	**-**	**-**	**-**	**-**	**+**	**+**	**+**	**+**	04	0.400 (0.000–0.960)	0.171 (0.000–0.540)	2.339
**3**	**+**	**+**	**+**	**-**	**-**	**-**	**-**	**-**	10	1.100 (0.160–2.030)	0.389 (0.000–0.940)	2.827
	**+**	**-**	**+**	**+**	**-**	**-**	**-**	**-**	01	0.100 (0.000–0.380)	0.456 (0.000–1.060)	0.219
	**+**	**-**	**-**	**+**	**-**	**-**	**+**	**-**	01	0.100 (0.000–0.380)	0.375 (0.000–0.920)	0.266
	**+**	**-**	**-**	**-**	**-**	**-**	**+**	**+**	01	0.100 (0.000–0.380)	0.373 (0.000–0.920)	0.086
**2**	**+**	**-**	**+**	**-**	**-**	**-**	**-**	**-**	09	1.000 (0.100–1.890)	1.161 (0.000–2.120)	0.861
	**+**	**-**	**-**	**+**	**-**	**-**	**-**	**-**	03	0.300 (0.000–0.790)	0.956 (0.080–1.830)	0.313
	**+**	**-**	**-**	**-**	**+**	**-**	**-**	**-**	01	0.100 (0.000–0.380)	0.951 (0.080–1.820)	0.105
	**+**	**-**	**-**	**-**	**-**	**+**	**-**	**-**	01	0.100 (0.000–0.380)	0.956 (0.080–1.830)	0.104
**1**	**+**	**-**	**-**	**-**	**-**	**-**	**-**	**-**	07	0.800 (0.000–1.590)	2.435 (1.050–3.810)	0.328
	**-**	**+**	**-**	**-**	**-**	**-**	**-**	**-**	08	0.900 (0.050–1.740)	2.435 (1.050–3.810)	0.369
	**-**	**-**	**+**	**-**	**-**	**-**	**-**	**-**	30	3.200 (1.620–4.770)	3.467 (1.820–5.100)	0.922
	**-**	**-**	**-**	**+**	**-**	**-**	**-**	**-**	17	1.800 (0.600–2.990)	2.854 (1.350–4.340)	0.630
	**-**	**-**	**-**	**-**	**+**	**-**	**-**	**-**	12	1.300 (0.280–2.310)	2.840 (1.340–4.330)	0.457
	**-**	**-**	**-**	**-**	**-**	**+**	**-**	**-**	18	1.900 (0.670–3.120)	2.855 (1.350–4.340)	0.665
	**-**	**-**	**-**	**-**	**-**	**-**	**-**	**+**	01	0.100 (0.000–0.380)	2.840 (1.340–4.330)	0.035
**0**	**-**	**-**	**-**	**-**	**-**	**-**	**-**	**-**	477	51.600 (47.110–56.080)	7.267 (4.930–9.590)	7.100

BMI: body mass index; WC: Waist circumference; WHtR: Waist to height ratio; TR SF: triceps skinfold; SE SF: subscapularis skinfold; SI SF: suprailiac skinfold; ΣTR + SE: sum of triceps and subscapularis skinfolds; ΣTR + SE + SI: sum of triceps, subscapular and suprailiac skinfolds;

+, presence of the anthropometric indicator; -, absence of the anthropometric indicator; O: Observed prevalence; E: Expected prevalence; O/E: Ratio between observed and expected prevalence; CI: Confidence interval.

Multinomial regression showed that at each 1 mL / kg / min increase in VO_2_max, the odds of adolescents having three, four, five or more anthropometric indicators of excess body fat decreased by 0.92, 0.85, and 0.73 times, respectively. Females were less likely of having three (OR = 0.39), four (OR = 0.17) and five or more (OR = 0.15) anthropometric indicators of excess body fat. In addition, adolescents with black / brown / yellow / indigenous skin color were twice as likely (OR = 2.28) of having simultaneity of four anthropometric indicators of excess body fat. Post-pubertal adolescents were more likely of having two (OR = 2.07) and five or more (OR = 1.70) simultaneous anthropometric indicators of excess body fat compared to the reference category (no anthropometric indicator of excess body fat index) (Table 5). Information stratified by sex can be seen in the Supplementary files (S1 Table).

**Table 5:** Association between anthropometric indicators and demographic, economic variables, physical activity, sexual maturation and VO_2_max in adolescents.

	VO_2_max	One indicator^a^		Two indicators^a^		Three indicators^a^		Four indicators^a^		Five or more indicators^a^	
	M±SD	n (%)	RC (95%CI)^b^	n (%)	RC (95% CI)^b^	n (%)	RC (95% CI)^b^	n (%)	RC (95% CI)^b^	n (%)	RC (95% CI)^b^
**Total**	38.80±5.83	94 (10,1)	0.98 (0.93–1.04)	46 (5.0)	0.93 (0.86–1.01)	44 (4.7)	**0.92 (0.85–0.99)**	40 (4.3)	**0.85 (0.78–0.93)**	227 (24.6)	**0.73 (0.69–0.78)**
**VO**_**2**_**max**											
**Sex**	**p<0.01**										
Males	42.68±5.34	43 (9,8)	1.00	23 (5.2)	1.00	24 (5.5)	1.00	25 (5.7)	1.00	102 (23.2)	1.00
Females	35.33±3.66	51 (10,5)	0.84 (0.45–1.58)	23 (4.7)	0.50 (0.21–1.16)	20 (4.1)	**0.39 (0.19–0.90)**	15 (3.1)	**0.17 (0.07–0.41)**	125 (25.6)	**0.15 (0.08–0.25)**
**Age**	**p = 0.55**										
14–16 years	38.70±5.59	44 (11,2)	1.00	22 (5.6)	1.00	17 (4.3)	1.00	21 (5.4)	1.00	98 (25.0)	1.00
17–19 years	38.94±6.14	50 (9,3)	1.49 (0.92–2.40)	24 (4.5)	1.54 (0.81–2.92)	27 (5.0)	0.91 (0.47–1.79)	19 (3.5)	1.56 (0.80–3.05)	129 (24.1)	1.15 (0.80–1.67)
**Skin color**	**p = 0.06**										
White	38.51±5.74	32 (9,4)	1.00	15 (4.4)	1.00	13 (3.8)	1.00	21 (6.2)	1.00	86 (25.2)	1.00
Brown/Black/Yellow/Indigenous	39.27±5.99	60 (10,5)	1.00 (0.61–1.63)	31 (5.4)	0.81 (0.41–1.62)	30 (5.2)	0.74 (0.36–1.50)	18 (3.1)	**2.28 (1.16–4.47)**	135 (23.6)	1.41 (0.96–2.05)
**Maternal education**	**p = 0.23**										
≥ 8 years of schooling	39.08±5.97	41 (10,3)	1.00	23 (5.8)	1.00	16 (4.0)	1.00	12 (3.0)	1.00	105 (26.4)	1.00
≤ 8 years of schooling	38.60±5.75	51 (9,8)	0.99 (0.61–1.60)	23 (4.4)	1.18 (0.62–2.25)	26 (5.0)	0.79 (0.40–1.55)	28 (5.4)	0.52 (1.25–2.08)	121 (23.4)	1.14 (0.79–1.65)
**Socioeconomic level**	**p = 0.04**										
High	38.95±5.83	57 (10,7)	1.00	28 (5.2)	1.00	28 (5.2)	1.00	20 (3.7)	1.00	131 (24.5)	1.00
Low	38.05±5.61	23 (9,2)	1.03 (0.58–1.85)	11 (4.4)	0.82 (0.35–1.87)	12 (4.8)	0.95 (0.44–2.07)	13 (5.2)	1.41 (0.64–3.09)	66 (26.3)	1.17 (0.75–1.80)
**Physical activity**	**p<0.01**										
Physically active	40.48±6.24	22 (10,4)	1.00	08 (3.8)	1.00	14 (6.6)	1.00	09 (4.3)	1.00	46 (21.8)	1.00
Little physically active	38.27±5.61	68 (9,9)	0.84 (0.48–1.47)	37 (5.4)	1.46 (0.61–3.46)	30 (4.3)	0.68 (0.33–1.43)	30 (4.3)	1.05 (0.45–2.45)	172 (24.9)	0.75 (0.48–1.18)
**Sexual maturation**	**p<0.01**										
Pre-pubertal / pubertal	39.23±5.89	65 (9,9)	1.00	28 (4.3)	1.00	33 (5.0)	1.00	26 (4.0)	1.00	140 (21.3)	1.00
Post-pubertal	37.70±5.60	28 (10,6)	1.41 (0.83–2.39)	18 (6.8)	**2.07 (1.06–4.03)**	11 (4.2)	1.20 (0.58–2.49)	14 (5.3)	1.82 (0.90–3.70)	84 (31.9)	**1.70 (1.14–2.51)**

OR, Odds Ratio; CI, Confidence Interval; M: mean; SD: standard deviation.

^a^ Reference category: zero anthropometric indicator of excess body fat.

^b^ Adjusted for all independent variables.

Binary regression showed that with each 1 mL / kg / min of VO_2_max increase, the odds of adolescents having the eight anthropometric indicators in excess were reduced by 0.55 times (Table 6). In addition, adolescents with black / brown / yellow / indigenous skin color were more likely (OR = 1.87) of having eight anthropometric indicators of excess body fat. Finally, females were less likely (OR = 0.02) of having the simultaneous presence of eight anthropometric indicators of excess body fat (BMI, WC, WHTr, TR SF, SE SF, SI SF, ΣTR + SE, ΣTR + SE + SI) when compared to males. Information stratified by sex can be seen in the Supplementary files (S2 Table).

**Table 6:** Odds ratios and 95% confidence intervals, crude and adjusted, between the simultaneous presence of eight anthropometric indicators of excess body fat and independent variables.

Variables	Crude analysis		Adjusted analysis	
	OR (CI95%)	p-value	OR (CI95%)	p-value
**VO**_**2**_**max**	0.78 (0.74–0.83)	**<0.01**	0.55 (0.48–0.63)	**<0.01**
**Sex**		0.74		**<0.01**
Males	1.00		1.00	
Females	1.07 (0.70–1.62)		0.02 (0.01–0.07)	
**Age**		0.40		0.66
14–16 years	1.00		1.00	
17–19 years	0.83 (0.55–1.27)		0.88 (0.49–1.56)	
**Skin color**		0.26		**0.02**
White	1.00		1.00	
Brown/Black/Yellow/Indigenous	0.78 (0.51–1.19)		1.87 (1.09–3.21)	
**Maternal education**		0.80		0.81
≥ 8 years of schooling	1.00		1.00	
≤ 8 years of schooling	1.05 (0.69–1.60)		0.93 (0.51–1.67)	
**Socioeconomic level**		0.25		0.44
High	1.00		1.00	
Low	0.76 (0.48–1.21)		1.26 (0.69–2.29)	
**Physical activity**		0.36		0.10
Physically active	1.00		1.00	
Little physically active	0.78 (0.47–1.31)		0.57 (0.29–1.11)	
**Sexual maturation**		**0.01**		
Pre-pubertal / pubertal	1.00		1.00	0.45
Post-pubertal	0.58 (0.37–0.91)		1.26 (0.68–2.34)	

OR, Odds Ratio; CI, Confidence Interval.

## Discussion

This study showed that the higher the VO_2_max of adolescents, the less chances of having three or more anthropometric indicators of excess body fat. In binary regression, the association among variables was reconfirmed, demonstrating that the higher the VO_2_max of adolescents, the lower the chances of having all (eight) anthropometric indicators of excess body fat. Other studies have shown that VO_2_max is inversely associated with body fat (regardless of method used to analyze body composition) and other cardiovascular risk factors in children and adolescents [19,20]. This fact can be justified by the action of VO_2_max on the inflammatory process caused by excess body fat.

Excess body fat is important inducer of systemic inflammation and this fact contributes to cardiovascular diseases linked to obesity [19]. Increased serum C-reactive protein (CRP) levels have been reported in obese individuals [21]. One of the most important and strong contributors to increased serum CRP levels is visceral adiposity [22], since adipokines stimulate the hepatic synthesis of CRP [19]. Studies have shown that CRP is positively associated with trunk adiposity measurements such as WC [23] and WHtR [24], which were analyzed in this study. Therefore, the greater the simultaneity of anthropometric indicators of excess body fat, the greater the fat concentration in the body, and this situation leads to increases in CRP concentrations.

As a consequence, CRP levels are closely linked to aerobic fitness levels [25]. A study has shown that the higher the aerobic fitness level, the lower the CRP level. The relationship between aerobic fitness and CRP is explained by the action that physical exercise exerts on adipose tissue, that is, the practice of physical exercise improves aerobic fitness and reduces the inflammatory process caused by body adiposity [26]. This improvement in aerobic fitness reduces inflammation of visceral adipose tissue, reduces adipocyte size, macrophage infiltration, increases peripheral blood flow, mitochondrial function, facilitates oxidation of fatty acids, decreases oxidative stress and improves resistance to cell stress [19].

This study used biological (sex, age, skin color, sexual maturation), economic (maternal schooling and economic level) and lifestyle aspects (physical activity) as control variables. Even with these variables, the inverse relationship between VO_2_max and body fat, investigated by clusters of anthropometric indicators, was maintained. This is in agreement with literature, which shows that regardless of any factors, low aerobic fitness is a risk factor for increased body fat levels and other health risk factors [3].

As a complementary result of this study, it was found that 10.1% of adolescents had only one anthropometric indicator above normal, 14% had two to four indicators above normal and 24.6% had five or more indicators above normal (and of these, 12% had eight anthropometric indicators in the classification of excess body fat). A study carried out with 627 Brazilian adolescents aged 14–17 years found a prevalence of 24.1% for the simultaneity of four anthropometric indicators at high levels (BMI, WC, sum of two and five skinfolds) [2]. The association of BMI, WC and sum of skinfolds is worrisome, since it simultaneously considers health risk conditions related to excess body fat, abdominal adiposity and excessive amount of fat in the central and peripheral regions of the body [2,27]. The result of this association leads to multiplicative deleterious effects on physiological parameters (such as iron deficiency, increased cardiovascular and metabolic risk), functional parameters (such as difficulty in locomotion, increase in precipitated fatigue and low levels of aerobic fitness) and psychosocial parameters (perception of barriers to physical activity practice) [19,20,28]. In this sense, intervening in subgroups that present this risk may be useful for modifying the health profile of school-age adolescents.

Studies that use the grouping of anthropometric indicators can help in the control of errors due to the low sensitivity of the method (such as BMI), and consequently attenuate the underestimation of these results [29]. More specific interventions can be performed when considering factors associated with clusters of indicators above normal [2,30], since there was a low prevalence of individuals with only one indicator above normal. In addition, by including factors that can be modified (such as VO_2_max), it is possible to elaborate VO_2_max improvement actions, predicting the changes that will occur in the different patterns of body fat distribution [31]. These data would have applicability not only for the prevention of diseases, but also in populations with diseases that cause changes in body composition [32].

The fact that VO_2_max was estimated by means of a submaximal test may be a study limitation, considering that the use of submaximal protocols to estimate VO_2_max has less precision than maximum protocols. However, submaximal tests are more practical to apply in samples with greater number of individuals [6]. In addition, submaximal indirect tests using heart rate may be ways to assess VO_2_max in adolescents with low physical fitness or who do not support maximal effort tests [27].

It could be concluded that as one VO_2_max unit of adolescents increased, the chances of simultaneously presenting three or more anthropometric indicators of excess body fat decreased, regardless of biological, economic and lifestyle factors. In addition, the present study identified that one in ten adolescents had all anthropometric indicators of excess body fat.

## Supporting information

S1 Table Association between anthropometric indicators and demographic, economic variables, physical activity, sexual maturation and VO_2_max in male and female. OR, Odds Ratio; CI, Confidence Interval; M: mean; SD: standard deviation. ^a^ Reference category: zero anthropometric indicator of excess body fat. ^b^ Adjusted for all independent variables.
(DOCX)

S2 Table Odds ratios and 95% confidence intervals, crude and adjusted, between the simultaneous presence of eight anthropometric indicators of excess body fat and independent variables according to sex. OR, Odds Ratio; CI, Confidence Interval.
(DOCX)

S1 File EXCEL. ORDER: Order Of Students
ID_QUIZ: Number of identification question in quiz
ID_SCHOOL: Number of identification school
SEX: female (number 2); male (number 3)
AGE: years
AGE_CATEGORIZED: 17–19 years (number 2); 14–16 years (number 3)
SKIN COLOR: brown / black / yellow / indigenous (number 2); White (number 3)
SCHOOL OF MOTHER: GREATER THAN 8 YEARS OF STUDY (number 2); LESS THAN 8 YEARS OF STUDY (number 3)
ECONOMIC STATUS: low class (number 2); high class (number 3)
ACTIVITY PHYSICAL: low active physically (number 2); physically active (number 3)
SEXUAL MATURATION: post puberal/puberal (number 2); pre puberal (number 3)
SUM 2: sum of triceps and subscapular folds
SUM 3: Sum of triceps and subscapular and suprailiaca folds
BODY MASS INDEX: overweight (number 1); normal weight (number 0)
WAIST: normal (number 0); excesso (number 1)
TRICEPS: normal (number 0); excesso (number 1)
SUBESCAPULAR: normal (number 0); excesso (number 1)
SUPRAILIACA: normal (number 0); excesso (number 1)
SUM_2: normal (number 0); excesso (number 1)
SUM_3: normal (number 0); excesso (number 1)
SIMULTANEOUS BEHAVIOUR: number of concurrent behaviors
STANDARD OF SIMULTANEITY: NUMBER OF EACH SIMULTANEOUS BEHAVIOUR
BEHAVIOUR FIVE OR MORE: ADOLESCENTS: 0 excess anthropometric indicator (number 0); 1 excess anthropometric indicator (number 1); 2 excess anthropometric indicators (number 2); 3 excess anthropometric indicators (number 3); 4 excess anthropometric indicators (number 4); 5 or more excess anthropometric indicators (number 5)
BINARY REGRESSION: 0 to 7 anthropometric indicators in excess (number 0); 8 anthropometric indicators in excess (number 1).
(XLSX)
