# Supplementary material for: Clusters of anthropometric indicators of body fat associated with maximum oxygen uptake in adolescents
Source: PLoS One. 2018 Mar 13;13(3):e0193965. doi: 10.1371/journal.pone.0193965 (PMC5849300; doi:10.1371/journal.pone.0193965)
Supplement: S1 Table — OR, Odds Ratio; CI, Confidence Interval; M: mean; SD: standard deviation. a Reference category: zero anthropometric indicator of excess body fat. b Adjusted for all independent variables. (DOCX) [file pone.0193965.s001.docx]

**Table 7.** Association between anthropometric indicators and demographic, economic variables, physical activity, sexual maturation and VO_2_max in male and female.

|  | MASCU **MASCULINO** | | | | | | | | | | | | |
| --- | --- | --- | --- | --- | --- | --- | --- | --- | --- | --- | --- | --- | --- |
|  | **VO_2_max One indicator^a^ Two indicators^a^ Three indicators^a^ Four indicator Five or more indicators^a^** | | | | | | | | | | | | |
|  | | M±SD | n  (%) | RC  (95%CI)^b^ | n  (%) | RC  (95% CI)^b^ | n  (%) | | RC  (95% CI)^b^ | n  (%) | RC  (95% CI)^b^ | n  (%) | RC  (95% CI)^b^ |
| **Total** | | 42.68±5.34 |  | 0.99 (0.92-1.06) |  | 1.00 (0.91-1.10) |  | | **0.86 (0.77-0.96)** |  | **0.87 (0.78-0.96)** |  | **0.74 (0.68-0.81)** |
| **Age** | | **p=0.45** |  |  |  |  |  | |  |  |  |  |  |
| 14-16 years | | 42.40±5.09 | 22 (9.0) | 1.00 | 11 (4.5) | 1.00 | 14 (5.7) | | 1.00 | 12 (5.9) | 1.00 | 55 (22.4) | 1.00 |
| 17-19 years | | 42.77±5.49 | 21 (10.8) | 1.45 (0.72-3.01) | 12 (6.2) | 1.10 (0.40-2.97) | 10 (5.1) | | 0.97 (0.35-2.48) | 13 (6.7) | 1.20 (0.48-3.03) | 47 (24.1) | 1.34 (0.76-2.39) |
| **Skin color** | | **p=0.20** |  |  |  |  | |  |  |  |  |  |  |
| White | | 42.38±5.01 | 29 (10.8) | 1.00 | 17 (6.3) | 1.00 | 19 (7.1) | | 1.00 | 08 (3.0) | 1.00 | 58 (21.6) | 1.00 |
| Brown/Black/Yellow/Indigenous | | 42.90±5.71 | 14 (8.6) | 0.81 (0.38-1.73) | 06 (3.7) | 0.39 (0.12-1.25) | 04 (2.5) | | 0.39 (0.12-1.22) | 16 (9.9) | **3.45 (1.36-8.76)** | 38 (23.5) | 1.46 (0.82-2.63) |
| **Maternal education** | | **p=0.36** |  |  |  |  |  | |  |  |  |  |  |
| ≥ 8 years of schooling | | 42.61±5.20 | 19 (9.8) | 1.00 | 12 (6.2) | 1.00 | 08 (4.1) | | 1.00 | 08 (4.1) | 1.00 | 46 (23.7) | 1.00 |
| ≤ 8 years of schooling | | 42.54±5.34 | 24 (10.0) | 0.81 (0.39-1.67) | 11 (4.6) | 0.91 (0.35-2.39) | 15 (6.3) | | 0.72 (0.28-1.84) | 17 (7.1) | 0.51 (0.19-1.34) | 55 (23.0) | 1.19 (0.67-2.11) |
| **Socioeconomic level** | | **p=0.40** |  |  |  |  |  | |  |  |  |  |  |
| High | | 42.77±5.17 | 27 (10.0) | 1.00 | 15 (5.6) | 1.00 | 15 (5.6) | | 1.00 | 14 (5.2) | 1.00 | 56 (20.8) | 1.00 |
| Low | | 42.04±5.56 | 10 (10.4) | 1.25 (0.51-3.06) | 04 (4.2) | 0.59 (0.14-2.43) | 07 (7.3) | | 1.56 (0.53-4.57) | 06 (6.3) | 1.04 (0.33-3.25) | 28 (29.2) | 1.19 (0.58-2.46) |
| **Physical activity** | | **p<0.01** |  |  |  |  |  | |  |  |  |  |  |
| Physically active | | 43.82±5.43 | 13 (10.8) | 1.00 | 02 (1.7) | 1.00 | 07 (5.8) | | 1.00 | 07 (5.8) | 1.00 | 23 (19.2) | 1.00 |
| Little physically active | | 42.02±5.12 | 28 (9.2) | 0.94 (0.44-2.01) | 20 (6.6) | 5.62 (1.22-25.81) | 17 (5.6) | | 1.24 (0.45-3.40) | 17 (5.6) | 1.03 (0.36-2.91) | 74 (24.3) | 0.84 (0.44-1.59) |
| **Sexual maturation** | | **p=0.07** |  |  |  |  |  | |  |  |  |  |  |
| Pre-pubertal / pubertal | | 42.79±5.31 | 27 (8.4) | 1.00 | 14 (4.3) | 1.00 | 19 (5.9) | | 1.00 | 15 (4.7) | 1.00 | 80 (24.8) | 1.00 |
| Post-pubertal | | 41.93±5.14 | 16 (14.2) | 1.73 (0.81-3.71) | 09 (8.0) | 2.59 (0.94-7.12) | 05 (4.4) | | 0.79 (0.27-2.31) | 10 (8.8) | 1.67 (0.65-4.29) | 19 (16.8) | **0.41 (0.20-0.86)** |
|  | |  |  |  |  | **FEMININO** |  | |  |  |  |  |  |
| **Total** | | 35.33±3.66 |  | 0.98 (0.90-1.08) |  | **0.82 (0.71-0.95)** |  | | 1.04 (0.91-1.18) |  | **0.79 (0.66-0.94)** |  | **0.70 (0.64-0.77)** |
| **Age** | | **p=0.30** |  |  |  |  |  | |  |  |  |  |  |
| 14-16 years | | 35.48±3.59 | 28 (9.6) | 1.00 | 13 (4.5) | 1.00 | 13 (4.5) | | 1.00 | 07 (2.4) | 1.00 | 74 (25.4) | 1.00 |
| 17-19 years | | 34.99±3.80 | 23 (11.7) | 1.47 (0.76-2.85) | 10 (5.1) | 1.25 (0.50-3.14) | 07 (3.6) | | 0.82 (0.30-2.19) | 08 (4.1) | 1.71 (0.58-4.98) | 51 (25.9) | 0.89 (0.53-1.51) |
| **Skin color** | | **p=0.03** |  |  |  |  |  | |  |  |  |  |  |
| White | | 35.02±3.65 | 31 (10.2) | 1.00 | 14 (4.6) | 1.00 | 11 (3.6) | | 1.00 | 10 (3.3) | 1.00 | 77 (25.2) | 1.00 |
| Brown/Black/Yellow/Indigenous | | 35.71±3.71 | 18 (10.1) | 1.29 (0.60-2.76) | 09 (5.0) | 1.43 (0.51-4.03) | 09 (5.0) | | 1.79 (0.62-5.17) | 05 (2.8) | 1.13 (0.32-3.98) | 48 (26.8) | 1.46 (0.81-2.61) |
| **Maternal education** | | **p=0.83** |  |  |  |  |  | |  |  |  |  |  |
| ≥ 8 years of schooling | | 35.29±3.79 | 22 (10.8) | 1.00 | 11 (5.4) | 1.00 | 08 (3.9) | | 1.00 | 04 (2.0) | 1.00 | 59 (28.9) | 1.00 |
| ≤ 8 years of schooling | | 35.27±3.61 | 27 (9.7) | 1.14 (0.58-2.23) | 12 (4.3) | 1.09 (0.40-2.94) | 11 (3.9) | | 0.73 (0.22-2.39) | 11 (3.9) | 0.31 (0.06-1.47) | 66 (23.7) | 0.80 (0.45-1.44) |
| **Socioeconomic level** | | **p=0.19** |  |  |  |  |  | |  |  |  |  |  |
| High | | 35.04±3.53 | 30 (11.3) | 1.00 | 13 (4.9) | 1.00 | 13 (4.9) | | 1.00 | 06 (2.3) | 1.00 | 75 (28.3) | 1.00 |
| Low | | 35.25±3.72 | 13 (8.4) | 0.86 (0.40-1.85) | 07 (4.5) | 0.98 (0.33-2.87) | 05 (3.2) | | 0.45 (0.13-1.54) | 07 (4.5) | 2.01 (0.61-6.65) | 38 (24.5) | 1.09 (0.60-1.97) |
| **Physical activity** | | **p=0.32** |  |  |  |  |  | |  |  |  |  |  |
| Physically active | | 35.38±3.53 | 09 (9.9) | 1.00 | 06 (6.6) | 1.00 | 07 (7.7) | | 1.00 | 02 (2.2) | 1.00 | 23 (25.3) | 1.00 |
| Little physically active | | 35.25±3.72 | 40 (10.4) | 0.71 (0.31-1.61) | 17 (4.4) | 0.51 (0.17-1.53) | 13 (3.4) | | **0.33 (0.12-0.89)** | 13 (3.4) | 1.03 (0.22-4.85) | 98 (25.5) | 0.59 (0.30-1.16) |
| **Sexual maturation** | | **p<0.01** |  |  |  |  |  | |  |  |  |  |  |
| Pre-pubertal / pubertal | | 34.53±3.67 | 38 (11.3) | 1.00 | 14 (4.2) | 1.00 | 14 (4.2) | | 1.00 | 11 (3.3) | 1.00 | 60 (17.9) | 1.00 |
| Post-pubertal | | 35.60±3.65 | 12 (8.0) | 1.08 (0.50-2.33) | 09 (6.0) | 2.27 (0.88-5.84) | 06 (4.0) | | 1.68 (0.61-4.66) | 04 (2.7) | 1.33 (0.40-4.43) | 65 (43.3) | **3.96 (2.34-6.70)** |

OR, Odds Ratio; CI, Confidence Interval; M: mean; SD: standard deviation.

^a^ Reference category: zero anthropometric indicator of excess body fat.

^b^ Adjusted for all independent variables.
