# Supplementary material for: Clusters of anthropometric indicators of body fat associated with maximum oxygen uptake in adolescents
Source: PLoS One. 2018 Mar 13;13(3):e0193965. doi: 10.1371/journal.pone.0193965 (PMC5849300; doi:10.1371/journal.pone.0193965)
Supplement: S2 Table — OR, Odds Ratio; CI, Confidence Interval. (DOCX) [file pone.0193965.s002.docx]

**Table 8.** Odds ratios and 95% confidence intervals, crude and adjusted, between the simultaneous presence of eight anthropometric indicators of excess body fat and independent variables according to sex.

|  | **MASCULINO** | | | |
| --- | --- | --- | --- | --- |
| **Variables** | **Crude analysis Adjusted analysis** | | | |
|  | **OR** **(CI95%)** | **p-value** | **OR (CI95%)** | **p-value** |
| **Age** |  | 0.79 |  | 0.66 |
| 14-16 years | 1.00 |  | 1.00 |  |
| 17-19 years | 0.92 (0.51-1.67) |  | 0.88 (0.49-1.56) |  |
| **Skin color** |  | 0.68 |  | **0.02** |
| White | 1.00 |  | 1.00 |  |
| Brown/Black/Yellow/Indigenous | 0.87 (0.47-1.62) |  | 1.87 (1.09-3.21) |  |
| **Maternal education** |  | 0.85 |  | 0.81 |
| ≥ 8 years of schooling | 1.00 |  | 1.00 |  |
| ≤ 8 years of schooling | 0.94 (0.52-1.71) |  | 0.93 (0.51-1.67) |  |
| **Socioeconomic level** |  | 0.14 |  | 0.44 |
| High | 1.00 |  | 1.00 |  |
| Low | 0.60 (0.30-1.18) |  | 1.26 (0.69-2.29) |  |
| **Physical activity** |  | 0.48 |  | 0.10 |
| Physically active | 1.00 |  | 1.00 |  |
| Little physically active | 0.78 (0.39-1.55) |  | 0.57 (0.29-1.11) |  |
| **Sexual maturation** |  | **0.02** |  |  |
| Pre-pubertal / pubertal | 1.00 |  | 1.00 | 0.45 |
| Post-pubertal | 2.74 (1.13-6.64) |  | 1.26 (0.68-2.34) |  |
|  | **FEMININO** |  |  |  |
| **Age** |  | 0.70 |  | 0.83 |
| 14-16 years | 1.00 |  | 1.00 |  |
| 17-19 years | 0.89 (0.52-1.54) |  | 1.08 (0.52-2.21) |  |
| **Skin color** |  | 0.20 |  | **0.03** |
| White | 1.00 |  | 1.00 |  |
| Brown/Black/Yellow/Indigenous | 0.70 (0.41-1.21) |  | 0.50 (0.26-0.95) |  |
| **Maternal education** |  | 0.44 |  | 0.21 |
| ≥ 8 years of schooling | 1.00 |  | 1.00 |  |
| ≤ 8 years of schooling | 1.24 (0.71-2.15) |  | 1.50 (0.78-2.90) |  |
| **Socioeconomic level** |  | 0.66 |  | 0.48 |
| High | 1.00 |  | 1.00 |  |
| Low | 0.87 (0.48-1.58) |  | 0.77 (0.38-1.57) |  |
| **Physical activity** |  | 0.60 |  | 0.81 |
| Physically active | 1.00 |  | 1.00 |  |
| Little physically active | 0.82 (0.40-1.70) |  | 1.12 (0.44-2.83) |  |
| **Sexual maturation** |  | **<0.01** |  | **0.01** |
| Pre-pubertal / pubertal | 1.00 |  | 1.00 |  |
| Post-pubertal | 0.37 (0.21-0.64) |  | 0.46 (0.24-0.88) |  |

OR, Odds Ratio; CI, Confidence Interval.
